# Single‐cell genomics based on Raman sorting reveals novel carotenoid‐containing bacteria in the Red Sea

**DOI:** 10.1111/1751-7915.12420

**Published:** 2016-10-17

**Authors:** Yizhi Song, Anne‐Kristin Kaster, John Vollmers, Yanqing Song, Paul A. Davison, Martinique Frentrup, Gail M. Preston, Ian P. Thompson, J. Colin Murrell, Huabing Yin, C. Neil Hunter, Wei E. Huang

**Affiliations:** ^1^ Department of Engineering ScienceUniversity of OxfordParks RoadOxfordOX1 3PJUK; ^2^ Leibniz Institute DSMZDeutsche Sammlung von Mikroorganismen und Zellkulturen GmbHInhoffenstrasse 7 B38124BraunschweigGermany; ^3^ Division of Biomedical EngineeringSchool of EngineeringUniversity of GlasgowGlasgowG12 8QQUK; ^4^ Department of Molecular Biology and BiotechnologyUniversity of SheffieldSheffieldS10 2TNUK; ^5^ Department of Plant SciencesUniversity of OxfordSouth Parks RoadOxfordOX1 3RBUK; ^6^ School of Environmental SciencesUniversity of East AngliaNorwichNR4 7TJUK

## Abstract

Cell sorting coupled with single‐cell genomics is a powerful tool to circumvent cultivation of microorganisms and reveal microbial ‘dark matter’. Single‐cell Raman spectra (SCRSs) are label‐free biochemical ‘fingerprints’ of individual cells, which can link the sorted cells to their phenotypic information and ecological functions. We employed a novel Raman‐activated cell ejection (RACE) approach to sort single bacterial cells from a water sample in the Red Sea based on SCRS. Carotenoids are highly diverse pigments and play an important role in phototrophic bacteria, giving strong and distinctive Raman spectra. Here, we showed that individual carotenoid‐containing cells from a Red Sea sample were isolated based on the characteristic SCRS. RACE‐based single‐cell genomics revealed putative novel functional genes related to carotenoid and isoprenoid biosynthesis, as well as previously unknown phototrophic microorganisms including an unculturable *Cyanobacteria* spp. The potential of Raman sorting coupled to single‐cell genomics has been demonstrated.

## Introduction

Microbes are the most diverse and abundant organisms on earth and play critical roles in biogeochemical carbon and nitrogen cycling (Paterson *et al*., 1997; Whitman *et al*., 1998; Schleifer, 2004; Huang *et al*., 2009a,b). However, the vast majority of microbes in natural environments have not yet been grown in the laboratory using traditional cultivation methods (Amann *et al*., 1995; Venter, 2003; Venter *et al*., 2004; Daniel, 2005; Swan *et al*., 2011; Rinke *et al*., 2013; Hedlund *et al*., 2014). Furthermore, to understand the function of microbes in a community, it is desirable to place even cultivable microorganisms in their ecological and environmental context instead of just studying pure cultures (Huang *et al*., 2015).

Metagenomics is a powerful cultivation‐independent approach for studying microbes. It can provide an overview of the diversity and metabolic blueprints of potential functions of microbes in environmental samples. Unfortunately, assembling individual discrete genomes from metagenomics data is quite difficult and rather costly, especially for samples with a high diversity of microorganisms. Although binning algorithms, which group contigs and assign DNA sequences to operational taxonomic units, have massively improved over the years, metagenomes assembled in this way may still be mosaics of DNA from different strains. Single‐cell genomics can complement metagenomics approaches and be used to establish genetic linkages between DNA sequences within individual cells (Swan *et al*., 2011; Lasken, 2012; Rinke *et al*., 2013). Although fluorescent‐activated cell sorting has been successfully used to sort single cells and subsequently perform single‐cell genomics (Rinke *et al*., 2013), Raman‐activated cell sorting (RACS) can provide a valuable alternative to sort cells based on single‐cell Raman spectra (SCRSs) which can reflect phenotypic and intrinsic biochemical fingerprints of cells (Huang *et al*., 2004, 2015). An SCRS is able to provide a label‐free biochemical profile of a single cell, which could contain information on nucleic acids, proteins, carbohydrates, lipids within the cell and specific Raman‐active compounds (e.g. carotenoids, isoprenoid, cytochrome *c*, poly‐β‐hydroxybutyrate and glycogen) (Li *et al*., 2012a,b,c; Zhang *et al*., 2015a,b). RACS‐mediated single‐cell genomics should, therefore, help to reveal phenotypic information and couple certain phenotypes to genotypes of cells.

**Figure 1:**
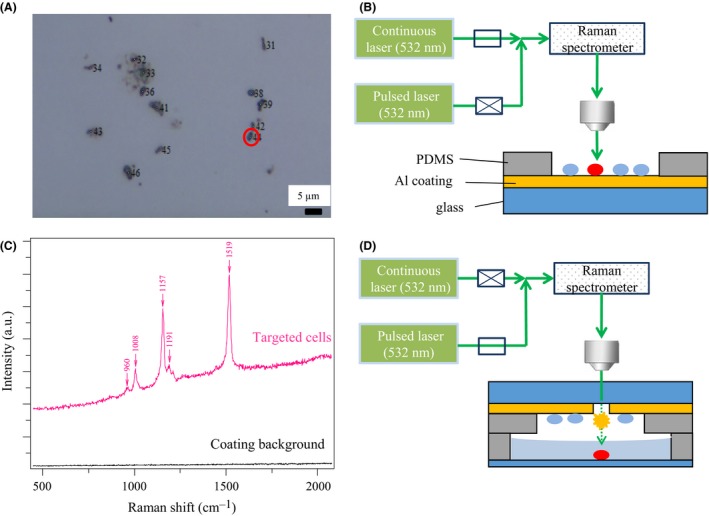
Illustration of resonant Raman‐activated single‐cell ejection (RACE). (A) Microscopic image of cells on a RACE chip. (B) The continuous laser is used for acquiring single‐cell Raman spectra. PDMS: Polydimethylsiloxane (C) Spectra of one single cell and the slide coating background. (D) The RACE chip is turned over and the target cell is ejected into the collector by a pulsed laser.

Raman‐activated cell sorting can be achieved by combining Raman single‐cell detection with optical tweezers (Huang *et al*., 2009a,b), microfluidic devices (Zhang *et al*., 2015a,b), or the so‐called Raman‐activated cell ejection (RACE) (Wang *et al*., 2013). Optical tweezers and microfluidic‐based RACS methods are, however, quite labour intensive and difficult to apply to complex samples. Previously, RACE has been performed by using two separate instruments: Raman and laser microdissection microscopes (Wang *et al*., 2013). In this study, we integrated Raman single‐cell detection and cell ejection into one system. This new system is now able to characterize single cells based on SCRS and accurately isolate cells of interest on a slide surface by employing laser‐induced forward transfer (LIFT) (Hopp *et al*., 2005). LIFT applies a laser to a thin‐layer‐coated surface which causes immediate and *local* evaporation of the coated layer, therefore pushing or ablating the selected cell on the layer into a collection device (Fig. 1).

Carotenoids are one of the most diverse chemicals in bacteria (Takaichi, 2008) and give strong and distinctive Raman spectra (Kochendoerfer *et al*., 1999; Krebs *et al*., 2003; Robert, 2009; Li *et al*., 2012a,b,c). Due to the resonance Raman effect, the Raman spectra of carotenoids are sharp and strong, providing typical and unambiguous carotenoid bands (Kochendoerfer *et al*., 1999; Krebs *et al*., 2003; Robert, 2009) making them ideal targets for the RACE system. Carotenoids are biologically important molecules, present in nearly all photosynthetic cells (Li *et al*., 2012a,b,c). For example, carotenoids are associated with both chlorophyll‐based photochemical reaction centres and rhodopsin‐based light‐activated proton pumps (Bryant and Frigaard, 2006), which are two important light‐harvesting (LH) systems of photosynthesis. In chlorophyll‐based LH systems, carotenoids absorb solar energy, quench free radicals and are structurally important parts of chlorophyll‐based LH complexes (Garcia‐Asua *et al*., 1998). In rhodopsin‐based LH systems, a carotenoid, namely β‐carotene, is a precursor of retinal, which is an essential component of bacteriorhodopsin and proteorhodopsin (Gonzalez *et al*., 2011). Hence, carotenoids are important indicative molecules related to light harvesting. Sorting carotenoid‐containing cells would, therefore, help to probe novel uncultured phototrophic bacteria. In this study, RACE was used to isolate carotenoid‐containing bacteria from the Red Sea. Both new photosynthetic bacteria and putative novel functional genes were discovered.

## Results

### Establishing the RACE system by integrating a cell ejection system with a Raman micro‐spectroscope

A 532 nm pulsed laser for single‐cell ejection has been integrated into a Raman micro‐spectroscope, which is designated as RACE (Fig. 1). Cells were added onto a RACE sampling chip (Fig. 1A and Fig. S1) and examined by the Raman micro‐spectroscope using a continuous 532 nm laser (Fig. 1B). SCRSs were used as sorting criteria to distinguish the phenotypic ‘profile’ of cells (in this case, the presence of carotenoids), and the positions of targeted cells were recorded (Fig. 1C). The RACE sampling chip was turned over, and single cells of interest were then ejected and harvested in a RACE collection chip (Fig. 1D and Fig. S2).

### Multiple displacement amplification from single cells isolated from RACE

Genomes of single or multiple (3–8) cells sorted with the RACE system were amplified by multiple displacement amplification (MDA; Blanco *et al*., 1989). As an initial test, *Escherichia coli* JM109 cells containing the plasmid p18GFP were used to examine the performance of single‐cell ejection. The green fluorescent protein gene *gfp* on the plasmid p18GFP and the universal stress protein A gene *uspA* (Chen and Griffiths, 1998) on the chromosome were used to assess gene recovery. The *gfp* on this plasmid has about 20–40 copies per cell, whereas *uspA* is a single copy gene on the *E. coli* chromosome. Figure S3A shows that the negative control did not contain amplified DNA, and three of seven samples contained MDA products from single *E. coli* cells. The other four samples did not yield visible MDA products (the products were too little to be seen in the gel), which may be due to the inefficient MDA of these cells. The PCR product of the *gfp* gene was recovered from all seven samples, while only one of the seven samples contained the *usp*A PCR product (Fig. S3B and S3C). This is probably due to amplification bias in the MDA, since multiple‐copy *gfp* genes have a better chance of being amplified than a single‐copy gene such as *usp*A in the one cell. Although sometimes the MDA yield was not high enough to be clearly viewed in agarose gel, the amount of DNA obtained was sufficient to perform PCR amplification (Fig. S3B and S3C). Nonetheless, this shows that it is possible that a single‐copy functional gene can be recovered by MDA from a single‐cell isolated by RACE.

### Sorting carotenoid‐containing bacteria from a Red Sea water sample based on their resonant Raman spectra

**Figure 2:**
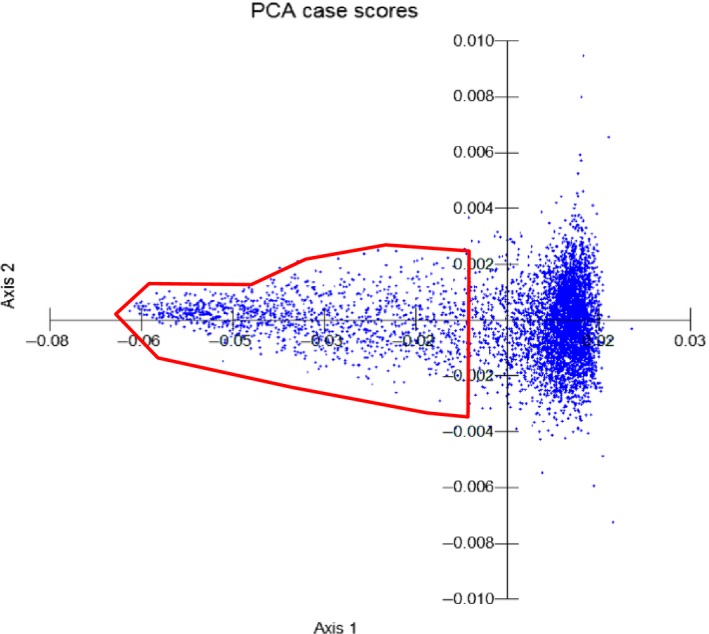
The PCA analysis of 5321 SCRS from the Red Sea sample. The group within red line area is cells containing various carotenoids, and the rest of cells have no carotenoids.

Raman‐activated cell ejection has been applied to sort bacterial cells sampled from surface seawater at Eilat on the Red Sea coast. SCRS of 5321 single cells have been obtained and analysed, and 33% of the analysed cells exhibited strong fluorescence and 67% had distinguishable SCRS for sorting. Figure 2 shows the principal component analysis (PCA) of those spectra, and the spots within the red line area are the cells containing carotenoids according to Raman spectra. The most significant loadings of PCA at axis 1 are 997–1007, 1145–1161 and 1503–1526 cm^−1^ (Fig. S4), corresponding to characteristic *v1*,* v2* and *v3* Raman bands of carotenoids (Robert, 2009; Li *et al*., 2012a,b,c). This is in a good agreement with the fact that carotenoid‐containing cells are distributed along axis 1 within the red line area (Fig. 2).

**Figure 3:**
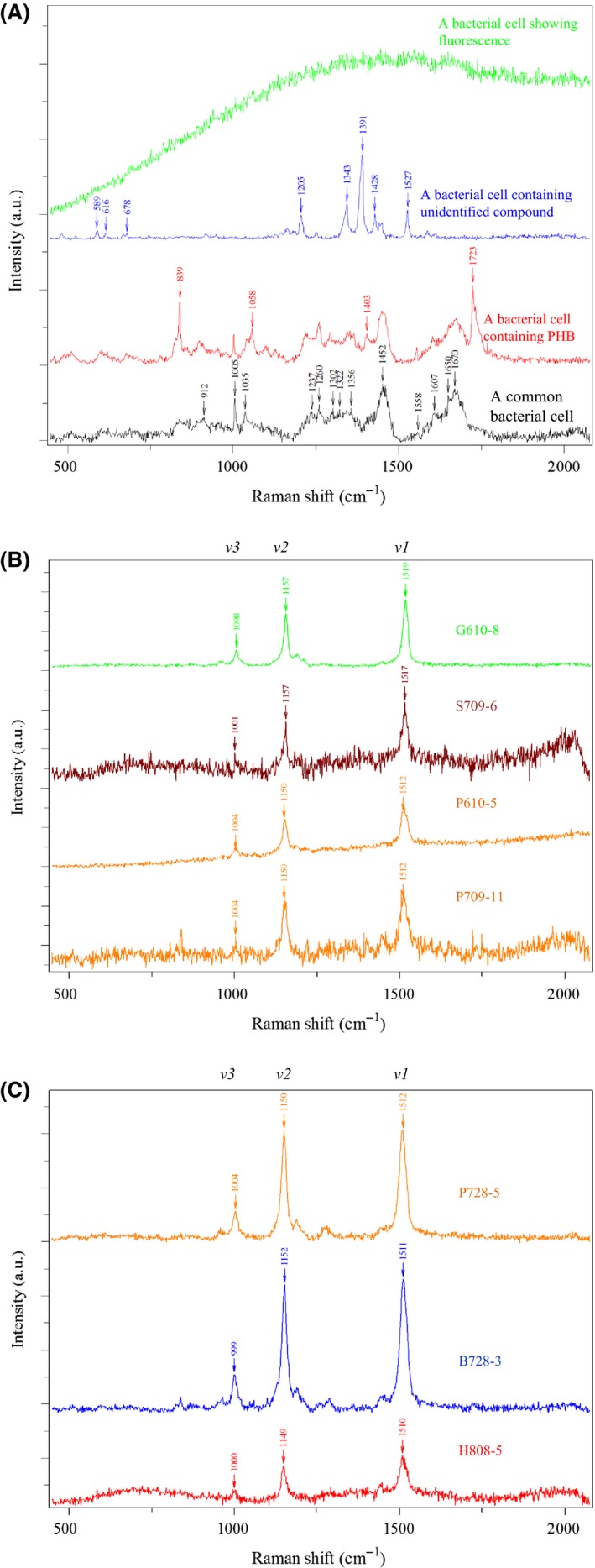
(A) Single cell Raman spectra of some typical bacteria in a Red Sea sample. (B) Raman spectra of four groups of ejected cells. Each group contains 3–8 cells which had the same carotenoid spectra. (C) Raman spectra of three groups of ejected individual cells. The positions of the carotenoid *v1*,* v2* and *v3* bands are labelled.

Figure 3A shows a few examples of SCRS, including a typical bacterial cell, individual cells showing fluorescence, cells containing poly‐β‐hydroxybutyrate (PHB) and unidentified compounds (cell images are shown in Fig. S5A). Since SCRS can reflect the biochemical phenotypes of cells (Huang *et al*., 2004, 2007a,b, 2009a,b; Li *et al*., 2012a,b,c; Berry *et al*., 2015), cells can be sorted based on the Raman biomarker bands of SCRS. For example, cells containing PHB have distinguishable Raman biomarkers (Majed and Gu, 2010) at 839, 1058, 1403 and 1123 cm^−1^ (Fig. 3A), which could be used to sort PHB containing cells. Interestingly, some SCRS also indicate that cells contain unidentified novel compounds, e.g. Fig. 3A shows a SCRS of a cell with a hydroxyisoquinoline‐like compound (Fig. S5A panel v). Since RACS is a new technology, a database with well‐characterized biomarkers is needed for the future analysis of cells.

In this study, the characteristic *v1, v2* and *v3* Raman bands of carotenoids were used as sorting criteria (Fig. 3). The *v1* band is the methyl rocking mode, and the *v2* and *v3* bands vary due to the different lengths of conjugated C=C bonds and stretching modes of C‐C bonds (Robert, 2009) in different carotenoids. Since the Raman spectral resolution is about 1 cm^−1^, variations in the positions of *v1, v2* and *v3* indicate the different structures of carotenoid presented in the cells. According to SCRS, 1223 (23%) cells contained carotenoids. Based on their carotenoid SCRS, 30 attempts of single‐cell ejection and whole genome amplification (WGA) were performed. Seven of the 30 attempts were successful in which 16S rRNA was recovered from the genome amplification. The SCRS of these seven successful cases are shown in Fig. 3B and 3C, and their information are listed in Table 1. Collectively these seven sorted samples could be resolved into five different types of carotenoids. Optical images of the sorted cells show that the cells had size of 0.3–1 μm (Fig. S5B).

**Table 1:** Summary of RACE‐isolated cells and WGA results

Sample name	P728‐5	B728‐3	H808‐5	S709‐6	P709‐11	G610‐8	P610‐5
Cells being ejected	1	1	1	3	5	8	8
Identification	*Pelomonas* spp.	*Bradyrhizobium* spp.	*Halomonas* spp.	*Shigella* spp.	*Pelomonas* spp.	*Cyanobacteria* spp.	*Pelomonas* spp.
Genome coverage (%)	8.18	6.65	4.17	10.74	6.9	8.95	19.29
Contamination	0	0		0	0	0	0
Putative genes for carotenoid biosynthesis	*crt*I	*isp*A, *crt*E, *crt*I	*shc* a	*crt*I	*idi*,* isp*A, *crt*E, *crt*I	*idi*	*crt*I, *crt*C, *crt*J
Putative genes for CO_2_ fixation	Aerobic‐type *cox*L/*cut*L;				CO_2_ concentrating mechanism/carboxysome shell protein; pepc		CO_2_‐fixation; Calvin‐Benson‐cycle related gene
Putative genes for haem/chlorophyll biosynthesis	*urod*,* cob*N, *chl*D, *ppox*,* fech*	*cob*N, haem biosynthesis	*cob*N	*cob*N, *uros, chl*I	*cob*N, *chl*I, *chl*D, *cpox*,* pbdg*,* ppox, fech*		*cob*N, *chl*D, *chl*I, haem oxygenase, *fech, pbdg, cpox*
Putative genes for fatty acid, alcohol, aldehyde metabolism	acc; Acyl‐CoA synthetases, short‐chain alcohol dehydrogenases, *mcat,* Molybdenum cofactor biosynthesis enzyme	Short‐chain alcohol dehydrogenases, short‐chain dehydrogenases, Acyl‐CoA synthetases, *fhl* subunit 4	Short‐chain alcohol dehydrogenases; Short‐chain dehydrogenases; Acyl‐CoA synthetases; *acc*; uncharacterized *fdh*	Acyl‐CoA synthetases; *hadh*;* acc*; short‐chain alcohol dehydrogenases; Predicted acyltransferases; *fhl* subunit 3 and 4, *mnh*D subunit; uncharacterized protein for *fdh* activity	Acyl‐CoA synthetases; Alcohol dehydrogenase, class IV; NAD+; uncharacterized protein for *fdh* activity	Alternatively TCA cycle; short‐chain alcohol dehydrogenases; Acyl‐CoA synthetases; Acyl‐CoA dehydrogenases; NAD+; Predicted acyltransferases; *fhl* subunit 3 and 4, *mnh*D subunit	Acyl‐CoA synthetases; *fhl* subunit 3 and 4, *mnh*D subunit

a The substrate of Shc (Squalene‐hopene cyclase) is a carotenoid compound.

*acc*, acetyl‐CoA carboxylase, carboxyltransferase component; *acp*, acyl carrier protein (used in FA synthesis); *chl*D, *chl*I: Mg‐chelatase subunit; *cob*N, cobalamin biosynthesis protein CobN and related Mg‐chelatases; *cox*L/*cut*L homologues, carbon monoxide dehydrogenase, large subunit homologues (used in carbon fixation via reductive acetyl‐CoA pathway); *cpox*, coproporphyrinogen III oxidase; *crt*C, hydroxyneurosporene synthase; *crt*E, geranylgeranyl pyrophosphate synthase; *crt*I, phytoene desaturase (dehydrogenase); *crt*J, CrtJ protein which is involved in spheroidene biosynthesis; *fdh*, formate dehydrogenase; *fech*, Protoheme ferro‐lyase (ferrochelatase); *fhl*, formate hydrogenase; *hadh*, 3‐hydroxyacyl‐CoA dehydrogenase; *idi*, isopentenyl diphosphate isomerase (or IPI, isopentenyl pyrophosphate isomerase); *isp*A, farnesyl diphosphate synthase; *mcat*, malonyl‐CoA:acyl carrier protein transacylase; *mnh*D, Na^+^/H^+^ antiporter subunit D; *pbgd*, Porphobilinogen deaminase; *pepc*, Phosphoenolpyruvate carboxylase; *ppox*, Protoporphyrinogen oxidase; *shc*, Squalene‐hopene cyclase; *urod*, Uroporphyrinogen‐III decarboxylase; *uros*, Uroporphyrinogen‐III synthase.

### Genome sequencing of isolated cells

**Figure 4:**
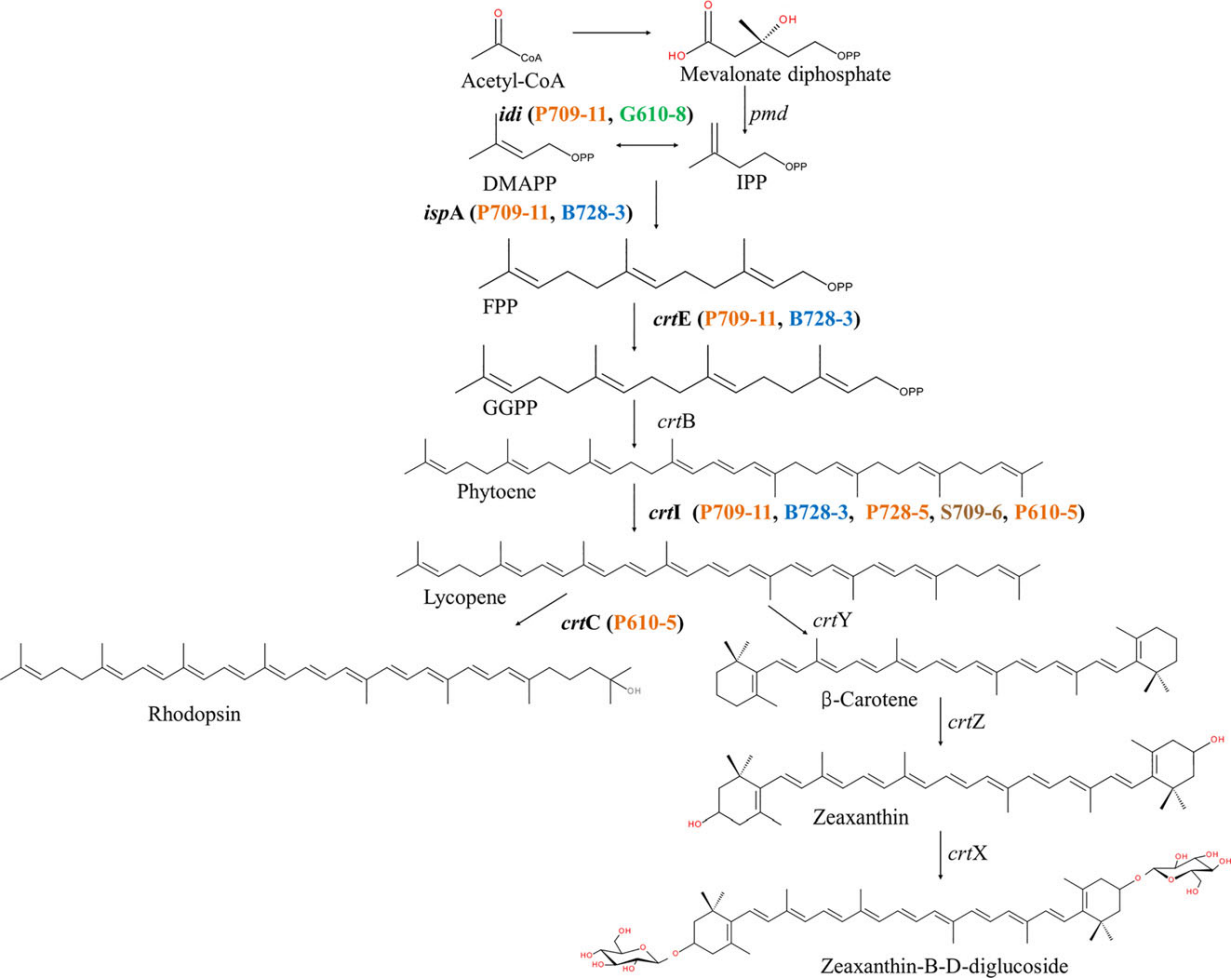
The identified genes encoding putative proteins in bacterial carotenoid synthesis pathway from Red Sea single cell(s) MDA. The ejected single cell(s) which contain the genes are bracketed and coloured. *idi*: isopentenyl diphosphate isomerase; *isp*A: farnesyl diphosphate synthase; *crt*E: Geranylgeranyl pyrophosphate synthase; *crt*B: phytoene synthase; *crt*I: phytoene desaturase (dehydrogenase); *crt*C: hydroxyneurosporene synthase; *crt*D: methoxyneurosporene dehydrogenase; *crt*F: hydroxyneurosporene‐O‐methyltransferase; *crt*Y: lycopene cyclase; *crt*Z: carotene hydroxylase; *pmd*: phosphomevalonate decarboxylase.

The estimated degree of genome completeness and putative contamination was based on universal marker genes analyses. The overall contamination based on CheckM analyses (Parks *et al*., 2015) was estimated to be relatively low in all cases (Table S3). However, sequence analyses also indicated relatively low genome coverage – less than 20% in all cases. The genome sequence data revealed functional genes related to carotenoid biosynthesis, CO_2_ fixation, haem/chlorophyll biosynthesis and fatty acid, alcohol and aldehyde metabolism. These data are summarized in Table 1. A detailed list of functional genes is given in Table S4. Despite the low estimated genome coverage, genes associated with carotenoid biosynthesis were identified in six of the seven sorted samples (P728‐5, B728‐3, P709‐11, S709‐6, G610‐8 and P610‐5). The recovered genes, which are directly involved in carotenoid biosynthesis pathways, are shown in Fig. 4. Specifically, isopentenyl diphosphate isomerase and geranylgeranyl pyrophosphate synthase (*isp*A) are involved in making the colourless substrate farnesyl pyrophosphate, and phytoene dehydrogenase (*crt*I) is responsible for synthesis of the red pigment lycopene (Fig. 4). H808‐5 contained two types of putative *shc* (squalene‐hopene cyclase) genes whose substrates are carotenoid compounds (Table 1), although no gene directly related to carotenoid synthesis was found in this sample (probably due to low genome coverage). These results validate the RACE method since the sorted cells were expected to contain carotenoids according to their SCRS.

**Figure 5:**
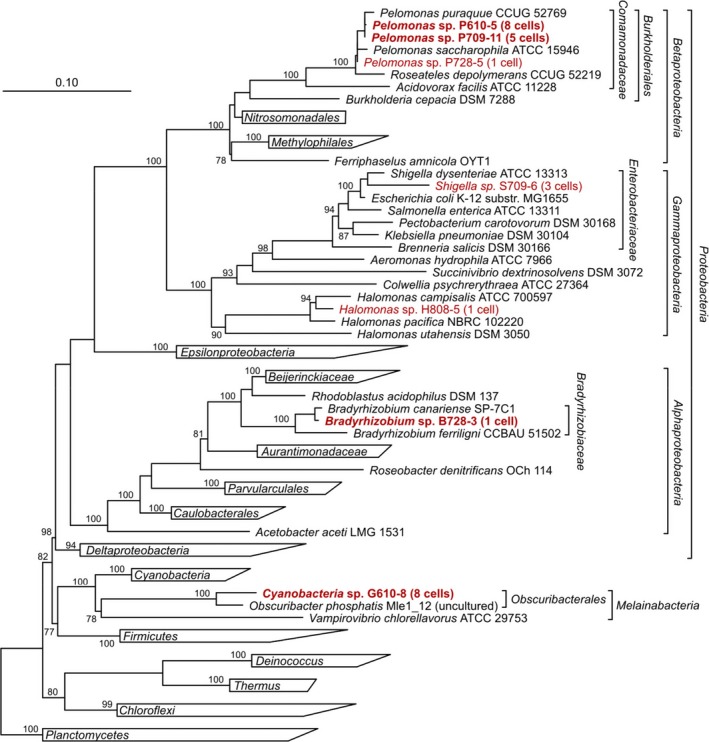
Neighbor joining tree indicating the phylogenetic relationships of cells sorted by Raman‐activated cell ejection and reference genomes. The tree is based on 16S rRNA gene sequences. Bootstrap support values above 75% are indicated at the respective nodes. Leaves referring to 16S rRNA gene sequences obtained from PCR products of MDA treated sorted cells, using universal bacterial primers, are marked in plain red lettering. Leaves referring to 16S rRNA gene sequences obtained from assembled single‐cell genome sequencing data of sorted cells are marked in bold red lettering. Black lettering indicates reference sequences.

A phylogenetic tree of the isolated samples is shown in Fig. 5. The carotenoid‐containing bacteria sorted from the Red Sea sample were broadly distributed over several different groups. According to 16S rRNA sequencing and BLAST (Altschul *et al*., 1990) against the NCBI bacterial 16S rRNA database, one of the sorted cells, G610‐8, is a new cyanobacterial order (GenBank accession no. KU667126), which gives a best hit (84% homology) to an uncultivated *Melainabacteria* spp. (Fig. 5). The 1.2 kb 16S rRNA sequencing has been independently repeated four times by three Sanger sequencing runs and one Illumina MiSeq sequencing run. All sequencing runs gave identical results, which rules out sequencing errors. This microorganisms is described as a novel, as yet uncultivated order of cyanobacteria (Soo *et al*., 2014).

## Discussion

The RACE technology has been applied to a Red Sea water sample to sort seven groups of cells and perform subsequent genome analyses (Table 1) and is a proof of concept for RACE applications in single‐cell genomics.

### MDA and genome recovery

There are further refinements needed to improve this technique in future studies, such as keeping the sorted cells viable, refining single‐cell lysis and genome amplification to increase genome coverage, and reducing contamination through better designed RACE chips. The low genome recovery is probably due to the following issues. (i) The cells may not be deposited into the lysis buffer in microwell (Fig. S1), the trajectory of cells can be further improved by adjusting the power of pulsed laser and exposure time. (ii) UV treatment of the Phi29 polymerase and inhibition residual hyperchloride used in the process, resulting in a loss of activity. Highly pure and DNA‐free Phi29 polymerase is now available in commercial market. (iii) The cells were dried on the chip for several weeks, and genome DNA may be damaged or degraded during the time. It can be improved by using fresh cells.

### Accurate single‐cell ejection

**Figure 6:**
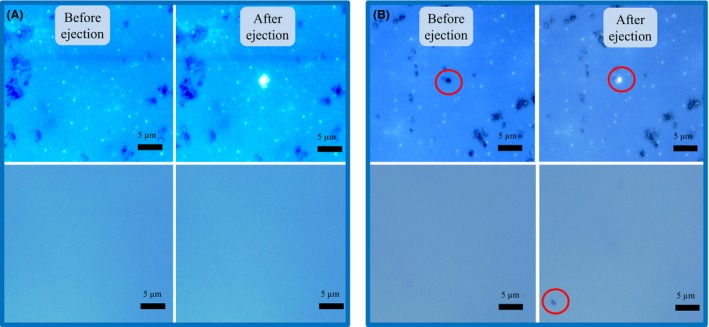
Microscopy images of the RACE chip (top row) which holds a sample from the Red Sea and the collector (bottom row) before and after applying the pulsed laser. (A) Pulsed laser focused at a position without cells. (B) Pulsed laser focused on a cell.

The thin coating material was able to absorb the energy of the 532 nm pulsed laser and to eject a single cell sitting on it (Fig. 6 and Fig. S2). Figure 6A and Fig. S2A show that the coating material got ablated after laser ejection, leaving a ~1.5 μm mark on the coating slide. No debris could be found on the collection chip after ejection in the blank controls, suggesting that the coating material was completely vapourized. Figure 6B and Fig. S2B indicate that single cells could be accurately isolated and collected by the RACE collection chip.

For single‐cell isolation using LIFT, the pulsed laser causes local vapourization of laser absorbing materials in the coating layer of the glass slide, which pushes the cells sitting on it to the collection well (Fig. 6 and Fig. S2). It has been shown that a 532 nm pulsed laser was able to vapourize metallic materials such as Cu and Ag (Bohandy *et al*., 1988). It has been previously demonstrated that cells can survive after isolation by LIFT, including bacterial cells (*E. coli*), various mammalian cells and sensitive mouse embryonic stem cells (Pique *et al*., 1999; Fernandez‐Pradas *et al*., 2004; Ringeisen *et al*., 2004; Hopp *et al*., 2005; Kattamis *et al*., 2007) (Ringeisen *et al*., 2002).

To achieve RACE, the thin metal coating in the chip should satisfy two criteria: (i) give a minimal Raman background (Fig. 1C), which ensures high‐quality SCRS for distinguishing phenotypes; (ii) be able to absorb a pulsed laser and cause *local* vapourization and expulsion of cells without thermal damage. After testing a variety of coating slides, a coating slide provided by Hesen Biotech (Shanghai, China) satisfied the above criteria as it has no Raman signal and absorbs a 532 nm pulsed laser causing ejection of cells.

### Discovering new genes using RACE

Three sorted samples (P728‐5, P709‐11 and P610‐5) with similar resonant Raman spectra (Fig. 3) were identified as closely related *Pelomonas* spp. according to phylogenetic analysis (Fig. S6A). Sequence identities in overlapping genome regions even indicate that these genomes may belong to the same species, but due to the low genome coverage resulting in only a few overlaps between the genomes, this could not be verified with confidence. *Pelomonas* is an understudied genus, and there are only a few reports concerning their metabolism, one suggesting that *Pelomonas* spp. could grow chemolithoautotrophically with hydrogen, and indicating the presence of CO_2_ fixation genes (Gomila *et al*., 2007). The sequences obtained from RACE‐based genomes of three *Pelomonas* spp. all included carotenoid and haem/chlorophyll biosynthesis and CO_2_ fixation genes, suggesting that these cells might be able to perform photosynthesis in their natural habitat, the Red Sea. Genes identified that are involved in CO_2_ fixation include phosphoenolpyruvate carboxylase (from P709‐11) which converts bicarbonate to oxaloacetate; a gene involved in making carboxysomes (for CO_2_ concentrating (from P709‐11); and a CO dehydrogenase that is involved in carbon fixation *via* the reductive acetyl‐CoA pathway (from P728‐5). It is possible that these cells are chemolithotrophs/oligotrophs and use these functional genes to survive in an oligotrophic system such as Red Sea.

A few genes encoding haem/chlorophyll biosynthesis enzymes were also recovered in the three *Pelomonas* spp., as well as in the *Bradyrhizobium* spp. B728‐3, *Halomonas* spp. H808‐5 and *Shigella* spp. S709‐6. Those genes included magnesium chelatase subunits, ferrochelatase and several enzymes upstream involved in protoporphyrin IX synthesis (Table 1 and summary of genome sequences). After performing a best match search against the NCBI GenBank database (Altschul *et al*., 1990), putative novel genes were found in the sorted samples. These genes have homology to well‐characterized genes but contain novel sequences. For example: a novel gene fragment (GenBank accession no. KX246394) encoding geranylgeranyl pyrophosphate synthase (CrtE) was found in *Bradyrhizobium* spp. B728‐3 (Fig. S7A); and a gene (GenBank accession no. KX246395) encoding CobN/magnesium chelatase from *Pelomonas* spp. P728‐5 is 87% homologous to the cobalamin gene *cob*N in *Bradyrhizobium* sp. S23321 DNA and 71% homologous to a gene encoding a CobN/magnesium chelatase in *Rhodobacter sphaeroides* (Fig. S7B). Alignment of two putative *shc* genes found in H808‐5 genomic sequence (Fig. S7C and D, GenBank accession no. KX246396 and KX246397) indicates that they are novel. SHC encoded by *shc* can catalyse very complex reactions, and any chemical mimicry of these reactions has so far been unsuccessful (Siedenburg and Jendrossek, 2011). Using single‐cell genomics, a link between genes and the identities of their host cells can be established, which may reveal the function of these microorganisms in their natural environment. This approach illustrates how genomic studies at the single‐cell level can provide information on unculturable and so far unknown microorganisms.

### RACE is able to distinguish cells with different Raman spectra and fluorescence

Raman micro‐spectroscopy is not only able to distinguish cells with different Raman spectra but also can be used to detect fluorescence at the single‐cell level which usually produces a large broad ‘bump’ spectrum dwarfing the Raman signal of SCRS (Fig. 3A). Fluorescence in some cells can be photo‐bleached by extending laser exposure time (Huang *et al*., 2007a,b). Since SCRS is independent of laser excitation, fluorescence interference in SCRS can be overcome by changing the incident lasers. SCRS are usually more informative than fluorescence. A RACE system able to distinguish and sort cells based on SCRS could, therefore, be more useful for the study of bacterial phenotypes.

## Conclusion

To our knowledge, this is the first report of the use of Raman‐activated cell sorting coupled to single‐cell genomics. Single‐cell genomics based on Raman sorting can not only sort cells with specific compounds (e.g. carotenoids in this case) but also isolate cells with carbon, nitrogen and general metabolic activity when it is coupled with stable isotope probing (Radajewski *et al*., 2000), which would help link the specific metabolism of single cells (e.g. carbon, nitrogen substrate metabolism or general metabolic activity) (Huang *et al*., 2004, 2007a,b; Li *et al*., 2012a,b,c; Wang *et al*., 2013; Berry *et al*., 2015) and assist in defining the ecological functions of uncultivated bacteria in the environment.

## Experimental procedures

### Chemicals, microorganisms and growth conditions

All chemicals used in this study were purchased from Sigma‐Aldrich (Dorset, UK) unless otherwise stated.

*Escherichia coli* JM109 (Promega, Southampton, UK) containing a p18GFP plasmid was incubated at 37°C in LB broth supplemented with 100 μg ml^−1^ ampicillin.

### Seawater samples

The seawater sample was collected from the pier of the Inter‐University Institute for Marine Sciences (IUI) in Eilat, Israel, on May 2015. The pier is 50 m long and the water depth is 5 m. The samples were concentrated 20 times using a Centricon Plus‐70 Ultracel PL‐100 (Merck Millipore, Tullagreen Carrigtwohill, Ireland) and then sent to the laboratory in Oxford on ice.

### Confocal Raman micro‐spectroscopy and spectral processing

Cells in concentrated seawater samples were washed before being analysed by Raman micro‐spectroscopy to remove salts that interfere with cell observation. Each cellular suspension (1 μl) was mounted in the designed mini‐wells of the RACE chip and allowed to air dry before Raman analysis (Fig. S1). The Raman spectra were acquired using a confocal Raman microscope (LabRAM HR Evolution; HORIBA Scientific, London, UK) equipped with an integrated microscope (BX41; Olympus, Essex, UK). A 50× long working distance objective (MPLFLN, NA 0.8; Olympus) was used to observe and acquire Raman signals from single cells and the overall magnification is 500. The laser beam position was calibrated and marked by the software Labspec6 (HORIBA Scientific). Cells were visualized by an integrated colour camera and a motorized XYZ stage (0.1 μm step). The Raman scattering was excited with a 532 nm Nd:YAG laser (Ventus, Laser Quantum, Manchester, UK). Raman measurements with grating 600 l mm^−1^ resulted in a spectral resolution of ∼1 cm^−1^ with 1019 data points. The laser power on a single cell was about 0.5 mW. The detector was a −70°C air‐cooled charge coupled device detector (Andor, Belfast, UK). LabSpec6 software was also used to control the Raman system and acquire the Raman spectra. Acquisition times for Raman spectra were 5 s for single‐cell measurements. The spatial location of each measured cell from the Red Sea sample were recorded in Labspec6 and used to identify cells for subsequent single‐cell ejection.

### Integration of single‐cell ejection into a Raman micro‐spectroscope

A 532 nm pulsed laser (ALPHALAS GmbH, Goettingen, Germany) was integrated into a confocal Raman microscope, sharing the same continuous laser (532 nm) light path and objective lens for SCRS measurement without changing mirrors and filters (Fig. 1).

The peak power was 1.1 kW, about two magnitudes lower than the traditional UV microdissection lasers (Table S1). The power of this 532 nm pulsed laser is maximally 3.4 mJ cm^−2^ (Table S1), which is much lower than the maximum resistible power (400 mJ cm^−2^) of the mirror and edge filters in the Raman microscope. An exposure test showed that the 532 nm pulsed laser did indeed cause no damage to the mirror and edge filters in this LabRAM HR Evolution Raman microscope (data not shown).

### Sterilization condition and reagents for single‐cell genomics

DNA contamination is a major issue in single‐cell genomics, due to the very small amount of template DNA derived from a single cell which has to be amplified by WGA. Standard sterilization practices are, therefore, not sufficient for single‐cell WGA and a modified WGA method had to be developed (Rinke *et al*., 2013, 2014) to minimize the influence of contaminant DNA. All the equipment and working surfaces were wiped with 10% (v/v) domestic bleach (Domestos, Surrey, UK). All the consumables were autoclaved and exposed to a UV light bulb (254 nm) inside a UV sterilized Laminar Flow cabinet for 1 h. All reagents were carefully sterilized, and the detailed process is described in Supplementary Information.

### Raman‐activated single‐cell ejection (RACE)

Supplementary information provides the details of reagent preparation and procedures. Briefly, 1 μl of the lysis buffer and 1 μl TE buffer were added into each well on the collection chip, which was then attached to the RACE chip. The enclosed chips were moved to the stage of the Raman microscope with the RACE chip facing down. The target cells were located by their coordinates and confirmed by comparing the bright‐field image taken during Raman spectra acquisition. The laser spot was manually aligned to the coating layer under the target cell. The target cells were ejected by evaporation of the coating layer upon application of a <1 s exposure of the 532 nm pulsed laser, and the cells were harvested in the collector. For a 12‐well RACE chip, at least two control wells were also set up: one negative control, which remained cell‐free, and one positive control, to which cell suspensions were added before WGA (see Data S1).

### Whole‐genome amplification on‐chip

The enclosed chips were carefully moved to a laminar flow chamber and the two chips were separated. A sterile coverslip was then placed on top of the collector chip. Wells were separated and independent with interference, and each well is a closed space. Cell lysis was carried out with lysis buffer (Qiagen, Manchester, UK) pre‐added in the collection chip (see Data S1). Three freeze‐thaw cycles were performed to enhance cell lysis. Subsequently, the chip was heated at 65°C for 10 min in a thermocycler (C1000; Bio‐Rad, Hemel Hempstead, UK) to ensure a complete lysis of cells. After adding 1 μl of Stop solution to neutralize the lysis buffer, 12 μl of reaction master mix (containing phi29 polymerase from Epicenter, Cambridge, Camlab, UK) was added to each well (Data S1). The collector chip was then covered by a coverslip and kept in the thermocycler at 30°C with the hot lid activated and set at 70°C. After incubation for 8 h, the Phi29 DNA polymerase was deactivated by heating to 65°C for 10 min. The MDA product was then transferred into sterilized 200 μl PCR tubes for storage.

### Library preparation and sequencing

Amplified DNA from single cells and cell consortia was quantified using a Qubit 1.0 fluorometer and a dsDNA HR assay kit (Life Technologies, Darmstadt, Germany). Libraries were constructed using a Nextera XT DNA Library Preparation Kit (Illumina, Cambridge, UK) and 1 ng of amplified input DNA, as per the manufacturer's instructions. Sequencing was done on an Illumina MiSeq machine with paired end settings and 301 cycles per read.

### Sequence processing and assembly

The raw sequences were subjected to adapter clipping and quality trimming using Trimmomatic (Bolger *et al*., 2014) with the following arguments: ‘LEADING:3 TRAILING:3 SLIDINGWINDOW:4:15 MINLEN:60’. Residual contaminant PhiX reads were removed from the datasets using FastQ Screen (http://www.bioinformatics.babraham.ac.uk/). In order to minimize subjective bias, no other potential contaminants from *E. coli* and human DNA were filtered from the datasets. To reduce MDA‐induced bias of overrepresented genome regions, the standard three‐step digital normalization protocol of the khmer suite was employed (Crusoe *et al*., 2015). The normalized reads were then assembled using Spades v3.6 (Bankevich *et al*., 2012).

### Sequence analyses

For each assembly, the genome completeness and degree of contamination was estimated using CheckM (Parks *et al*., 2015). Prokka v1.10 was used for gene prediction and annotation. Universal marker gene products were extracted using fetchMG (http://www.bork.embl.de/software/mOTU/fetchMG.html), compared against the NCBI nr database using BLAST and phylogenetically classified using MEGAN5 (Huson and Weber, 2013). For 16S rRNA gene sequences extracted from assembled genomes or generated from screening PCRs, the rough taxonomical placement was determined based on BLAST searches against the NCBI bacterial 16S RNA database, and suitable reference sequences originating from described type strains were selected accordingly. All 16S rRNA sequences were aligned against the SILVA database using Sina (Pruesse *et al*., 2012) and incorporated into phylogenetic trees using Arb v5.5 (Ludwig *et al*., 2004). Only sequences longer than 1200 bp were used for calculation of the basic tree backbones, while sequences shorter than 1200 bp were subsequently added using the parsimony function of Arb.

## Conflict of interest

The authors declare no conflict of interest.

## Supporting information


**Data S1.** Materials and methods.
**Fig. S1.** Illustration of the RACE chip design.
**Fig. S2.** Isolation of a single *E. coli* cell using the RACE chip.
**Fig. S3.** (A) Agarose gel image of multiple displacement amplifications (MDAs) showing high‐molecular‐weight DNA.
**Fig. S4.** PCA axis 1 loadings.
**Figure S5.** (A) Microscopy image of cells in Fig. 2A and B.
**Fig. S6.** (A) Phylogenetic tree of three *Pelomonas* spp. P728‐5, P709‐11 and P610‐5 isolated in this study.
**Fig. S7.** (A) The partial gene encoding CrtE from *Bradyrhizobium* spp. B728‐3 (1 cell) is novel and related to *crtE* genes from Cyanobacteria *Synechocystis* spp. and *Gloeobacter* spp. according to BLAST analysis.
**Table S1.** Pulsed laser power comparison for cell isolation.
**Table S2.** Primers used in this study.

**Table S3.** Genome coverage and contamination assessment.

**Table S4.** Summary of genome sequences.
