# Supplementary material for: Single‐cell genomics based on Raman sorting reveals novel carotenoid‐containing bacteria in the Red Sea
Source: Microb Biotechnol. 2016 Oct 17;10(1):125–37. doi: 10.1111/1751-7915.12420 (PMC5270752; doi:10.1111/1751-7915.12420)
Supplement: Supplementary file 1 — Data S1. Materials and methods. Fig. S1. Illustration of the RACE chip design. Fig. S2. Isolation of a single E. coli cell using the RACE chip. Fig. S3. (A) Agarose gel image of multiple displacement amplifications (MDAs) showing high‐molecular‐weight DNA. Fig. S4. PCA axis 1 loadings. Figure S5. (A) Microscopy image of cells in Fig. 2A and B. Fig. S6. (A) Phylogenetic tree of three Pelomonas spp. P728‐5, P709‐11 and P610‐5 isolated in this study. Fig. S7. (A) The partial gene encoding CrtE from Bradyrhizobium spp. B728‐3 (1 cell) is novel and related to crtE genes from Cyanobacteria Synechocystis spp. and Gloeobacter spp. according to BLAST analysis. Table S1. Pulsed laser power comparison for cell isolation. Table S2. Primers used in this study. [file MBT2-10-125-s001.pdf]

Supporting information

Materials and methods

Fabrication of sampling and collection chips for single cell genomics

Sampling and collection RACE chips designed for single cell genomics are illustrated in Fig. S1. To make collection RACE chips, multi-layered Polydimethylsiloxane (PDMS) consisting of two concentric wells were fabricated. A PDMS layer (3 mm thick) was made by pouring a mixture of PDMS base and curing agent (Sylgard 184, Dow Corning) at a ratio of 10:1 (w/w) onto a silanised wafer in a container, followed by curing at 65°C overnight. Small wells with 3.5 mm diameter were then punched in the PDMS layer (Fig. S1). The second PDMS layer (0.5 mm thick) was made by spinning PDMS prepolymer on a silanised wafer at 200 rpm for 30 s, followed by curing at 65 ^{o}C for 1 h (Fig. S1). The two PDMS layers were bonded together using oxygen plasma treatment, and then 2 mm diameter holes were punched in the 0.5 mm thick PDMS layer concentrically to the 3.5 mm holes (Fig. S1). The resultant PDMS well block was irreversibly bonded onto a coverslip using oxygen plasma treatment. To make sampling RACE chips, single-layered 0.5 mm thick PDMS wells with 1 mm diameter were manufactured upon thin layer coating slides in a similar manner (Fig. S1).

Slides with various coating materials were purchased from Hesen Biotech (Shanghai, China) for testing. The layer coating had to be less than 100 nm to ensure transparency. Various coating materials including PEN (polyethylene naphthalate), PET (polyethylene terephthalate) and POL (polyester), Hesen001, Hesen002 and Hesen003 have been tested to achieve RACE. A 532-nm pulsed laser (0.95 ns FWHM, Emax = 4.3 µJ, rep rate =0.1–16.6 kHz) was found to achieve single cell ejection on PEN, PET, POL, metal-coated slides. Among these coating materials, Hesen003 resulted in good quality SCRS with minimal Raman background signal (Fig. 1C).

Chip design for accurate sorting of single bacterial cells

One of the major challenges of single cell genomics is contamination. *E. coli* with p18GFP was used to test the performance of single cell sorting by RACE. Some marker genes, including 16S rRNA, *gfp* and *usp*A for *E. coli* containing plasmid p18GFP, were recovered from the products of WGA (Fig. S3).

Sterilisation condition and reagents for single cell genomics

Single cell genomics (SCG)-grade water:

Ultrapure nuclease-free water was exposed to UV light in petri-dishes for 16 hours before being used to make reagents and solutions.

Lysis buffer D2:

The lysis buffer was prepared according to the manual (single cell kit, Qiagen, UK). 500µl of SCG-grade water and 45.5 µl of 1M DTT were added to buffer DLB (single cell kit, Qiagen, UK) and aliquoted into UV sterilised Eppendorf tubes.

Tris-EDTA buffer and DMSO:

Tris-EDTA (pH=8) buffer and DMSO were filtered through a 0.2 µm filter and aliquoted into UV sterilised Eppendorf tubes.

Random Hexamer:

Random hexamers were synthesised at Integrated DNA Technologies (IDT) and diluted to 500 µM in SCG-grade water.

Reaction buffer:

The reaction buffer contained 1x Repliphi29 reaction buffer (Epicentre, US), 50µM random hexamers, 5% DMSO, 10mM DTT, dNTPs (0.4mM) and aliquoted into UV sterilised Eppendorf tubes.

DNA polymerase:

The phi29 polymerase was purchased from Epicentre (Epicentre, US) and aliquoted into UV sterilised Eppendorf tubes.

Collector chip

The collector chip was soaked in 1.5% hypochlorite solution for 10 minutes and then washed with ultrapure water thoroughly to remove excessive hypochlorite. The collector chip was exposed to UV light for 60 mins and then stored in a sealed container. Glass cover-slips (20x60mm) were treated and stored in the same way.

UV sterilization before WGA

The lysis buffer D2, TE buffer, Stop solution (Qiagen Co, UK), reaction buffer and DNA polymerase phi29 were thawed (the DNA polymerase must be thawed on ice). The reaction buffer and phi29 were mixed at a volume ratio of 11:1 to make a reaction master mix (RMM). The EP tubes containing the reagent were placed in a reflection chamber filled with 4 ^{o}C water and were exposed to UV (254 nm) for 40-60min.

Reference

1. **Marchesi JR, Sato T, Weightman AJ, Martin TA, Fry JC, Hiom SJ, Wade WG.** 1998. Design and evaluation of useful bacterium-specific PCR primers that amplify genes coding for bacterial 16S rRNA. Applied and Environmental Microbiology **64:**795-799.
2. **Manefield M, Whiteley AS, Griffiths RI, Bailey MJ.** 2002. RNA stable isotope probing, a novel means of linking microbial community function to Phylogeny. Applied and Environmental Microbiology **68:**5367-5373.
3. **Uchiyama T, Abe T, Ikemura T, Watanabe K.** 2005. Substrate-induced gene-expression screening of environmental metagenome libraries for isolation of catabolic genes. Nature Biotechnology **23:**88-93.
4. **Chen J, Griffiths MW.** 1998. PCR differentiation of Escherichia coli from other Gram-negative bacteria using primers derived from the nucleotide sequences flanking the gene encoding the universal stress protein. Letters in Applied Microbiology **27:**369-371.

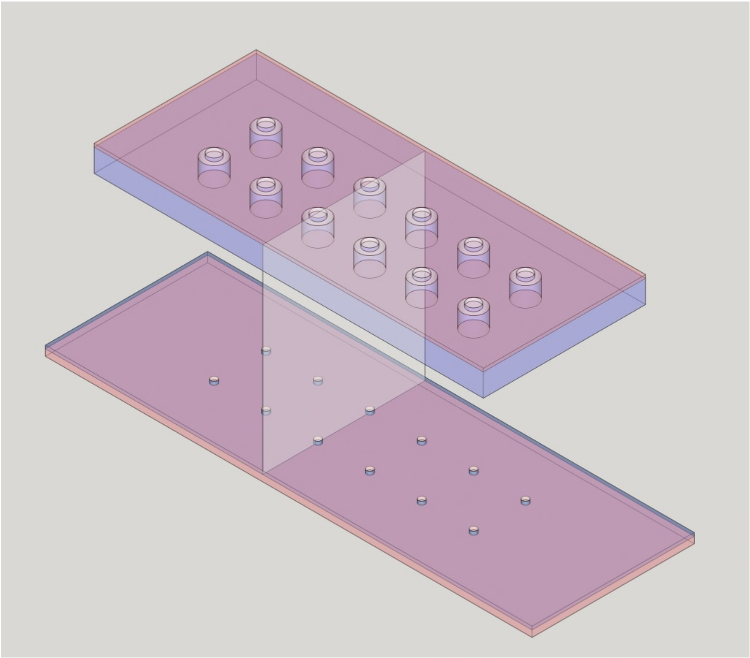

Figure S1. Illustration of the RACE chip design. (A) Polydimethylsiloxane (PDMS) (blue) was casted on a coated microscopic slide to create small wells on both sampling chip (bottom) and collection chip (top).

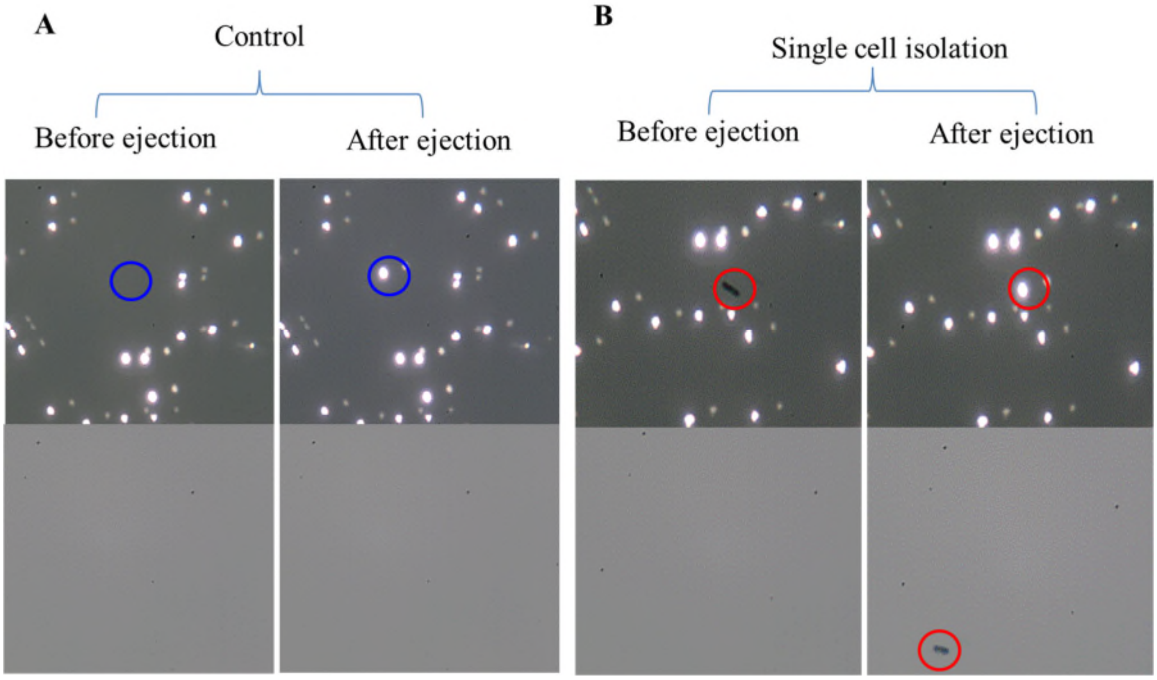

Figure S2. Isolation of a single *E. coli* cell using the RACE chip. Microscopic images of the RACE sampling (top row) and collection chips (bottom row) before and after applying the pulsed laser for single cell isolation. (A) Blank slide control (B) single *E. coli* ejection.

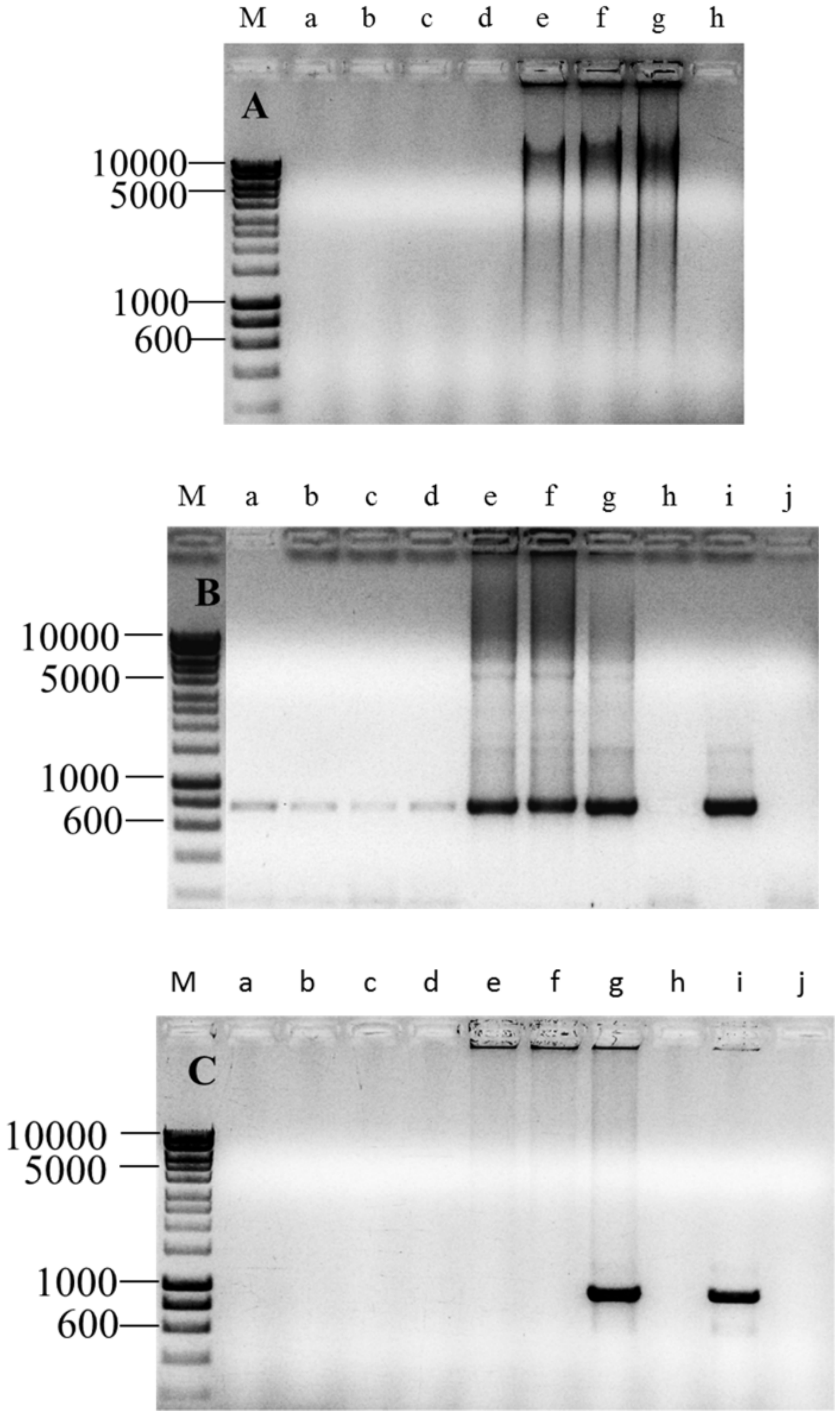

Figure S3. (A) Agarose gel image of multiple displacement amplifications (MDAs) showing high molecular weight DNA. **(B)** Agarose gel image of the PCR products of *gfp* gene and **(C)** *usp*A gene from isolated single *E. coli* cells. Lane M, DNA ladder; a – g, one ejected cell in each sample; h, negative control (no cell was ejected); i, positive control for PCR; j, negative control for PCR.

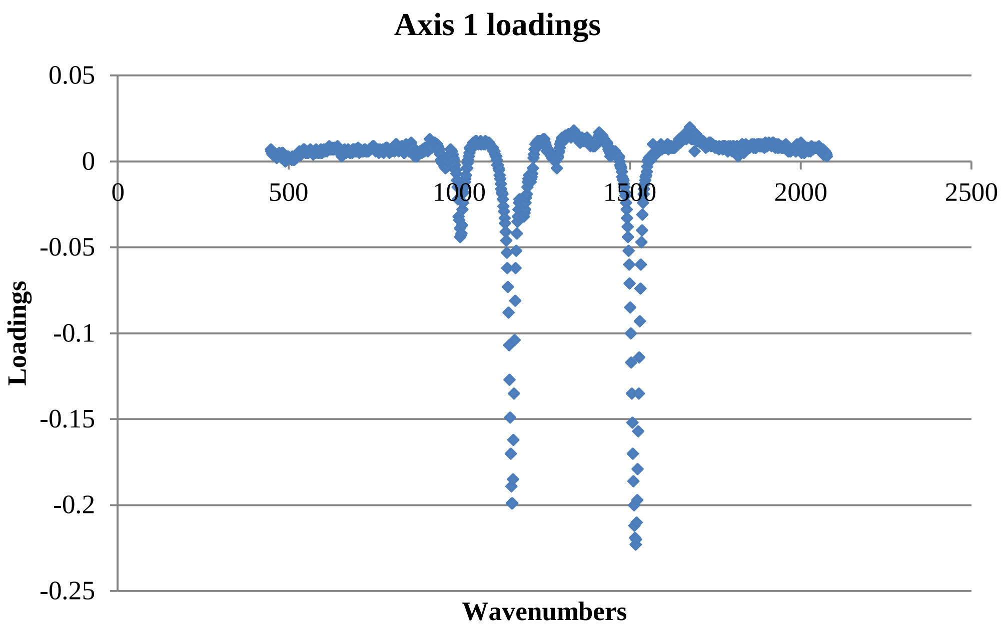

Figure S4. PCA axis 1 loadings. The predominant loadings at axis 1 are 997-1007, 1145-1161 and 1503-1526 cm^{-1}, corresponding to characteristic *v1*, *v2* and *v3* Raman bands of carotenoids.

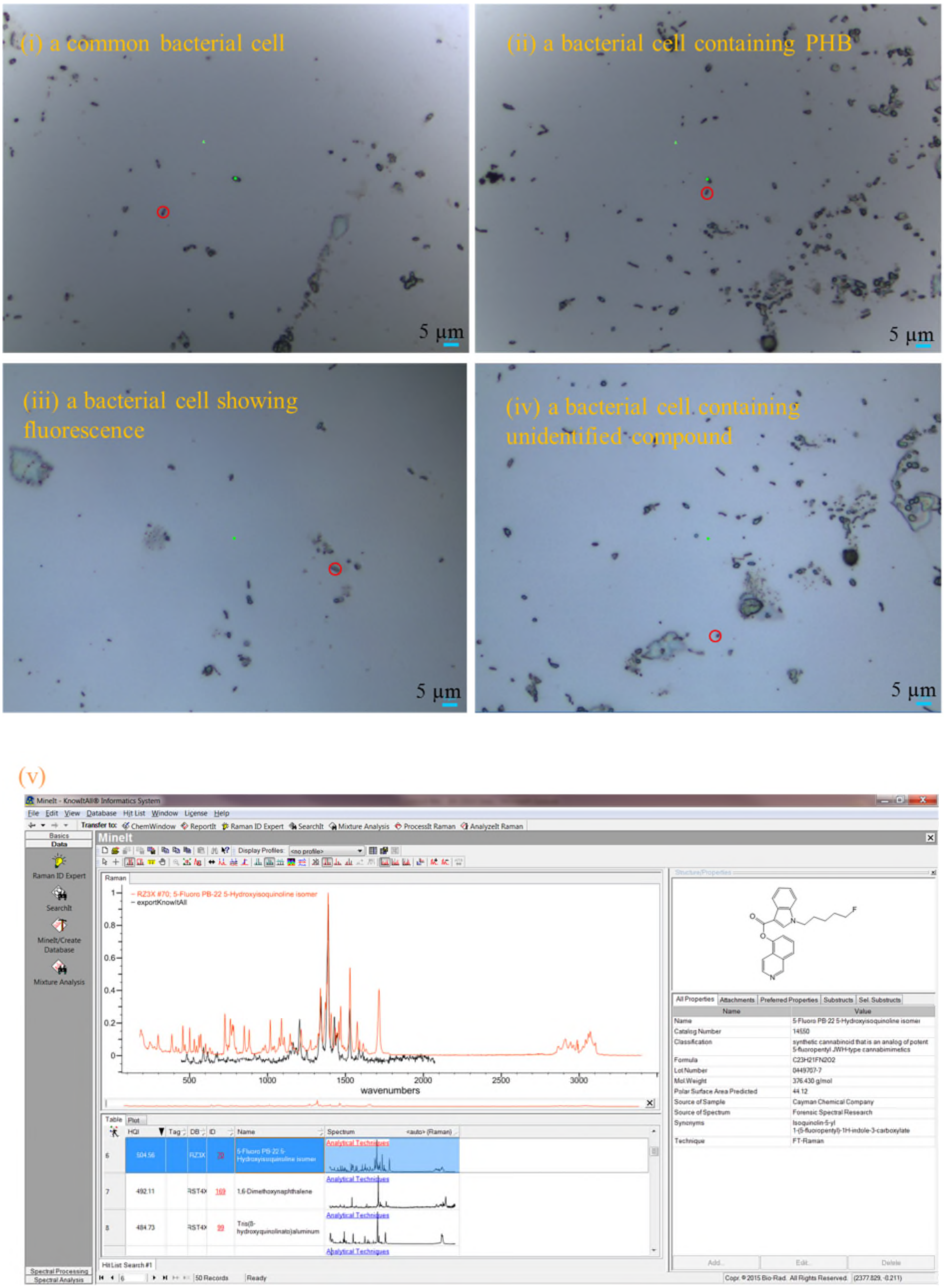
(i) a common bacterial cell
5 µm
(ii) a bacterial cell containing PHB
5 µm
(iii) a bacterial cell showing fluorescence
5 µm
(iv) a bacterial cell containing unidentified compound
5 µm
(v)
MineIt - KnowItAll® Informatics System
MineIt
RZ3X #70: 5-Fluoro PB-22 5-Hydroxyisoquinoline isomer
exportKnowItAll
wavenumbers
Raman ID Expert
SearchIt
MineIt/Create Database
Mixture Analysis
Copr. © 2015 Bio-Rad. All Rights Reserved.

(A)

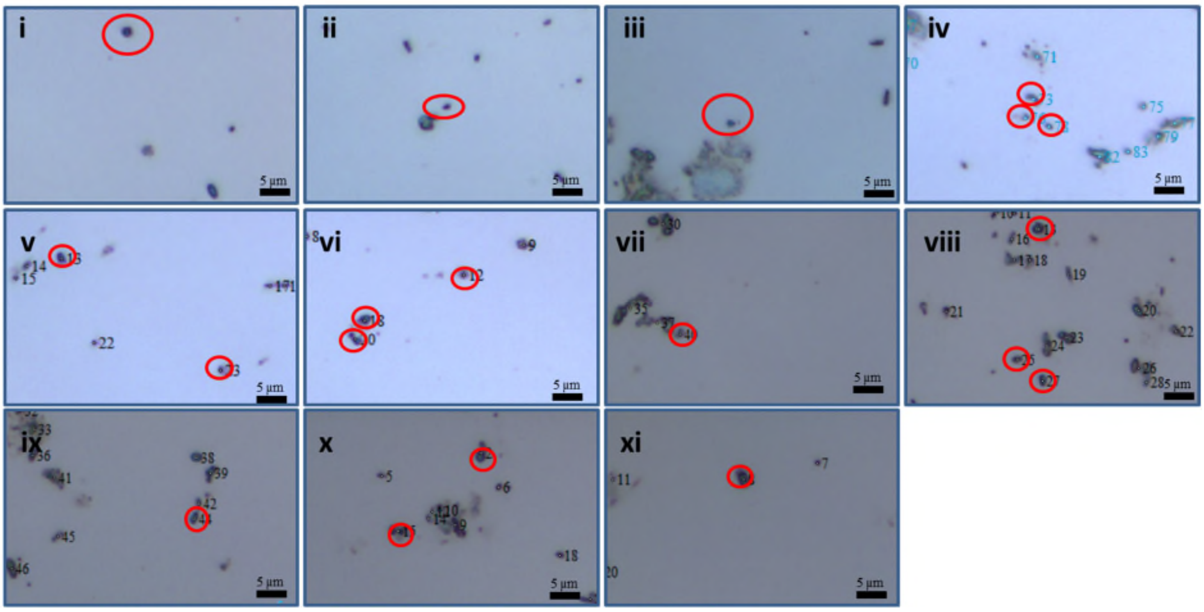

(B)

Figure S5. (A) Microscopy image of cells in Figure 3A and 3B. (i) a common bacterial cell; (ii) a bacterial cell containing PHB; (iii) a bacterial cell showing fluorescence; (iv) a bacterial cell containing (an) unidentified compound(s). The target cells are circled. The scale bar at the right bottom is 5 µm. (v) Matching SCRS of (iv) against database indicates that this cell may contain a hydroxyisoquinoline-like compound. **(B)** Microscopy images of ejected cells listed in Table 1. Ejected cells are circulated. (i) H808-5, (ii) B728-3, (iii) P728-5, (iv) S709-6, (v, vi) P709-11, (vii, viii, ix, x, xi) G610-8.

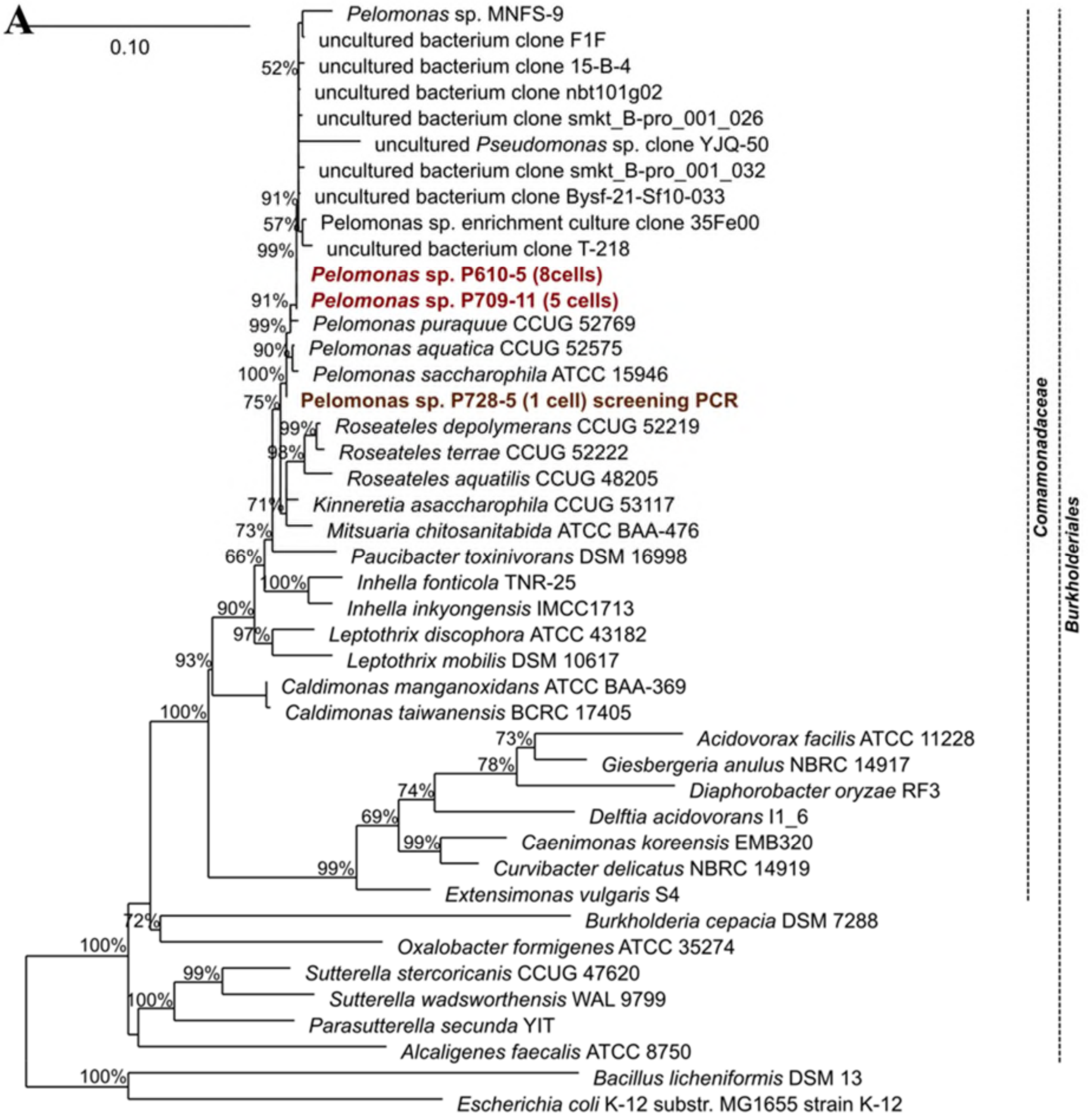
A
0.10
Pelomonas sp. MNFS-9
uncultured bacterium clone F1F
52%
uncultured bacterium clone 15-B-4
uncultured bacterium clone nbt101g02
uncultured bacterium clone smkt_B-pro_001_026
uncultured Pseudomonas sp. clone YJQ-50
uncultured bacterium clone smkt_B-pro_001_032
91%
uncultured bacterium clone Bysf-21-Sf10-033
57%
Pelomonas sp. enrichment culture clone 35Fe00
99%
uncultured bacterium clone T-218
Pelomonas sp. P610-5 (8cells)
91%
Pelomonas sp. P709-11 (5 cells)
99%
Pelomonas puraquue CCUG 52769
90%
Pelomonas aquatica CCUG 52575
100%
Pelomonas saccharophila ATCC 15946
75%
Pelomonas sp. P728-5 (1 cell) screening PCR
99%
Roseateles depolymerans CCUG 52219
98%
Roseateles terrae CCUG 52222
Roseateles aquatilis CCUG 48205
71%
Kinneretia asaccharophila CCUG 53117
73%
Mitsuaria chitosanitabida ATCC BAA-476
66%
Paucibacter toxinivorans DSM 16998
100%
Inhella fonticola TNR-25
90%
Inhella inkyongensis IMCC1713
97%
Leptothrix discophora ATCC 43182
93%
Leptothrix mobilis DSM 10617
Caldimonas manganoxidans ATCC BAA-369
100%
Caldimonas taiwanensis BCRC 17405
73%
Acidovorax facilis ATCC 11228
78%
Giesbergeria anulus NBRC 14917
Diaphorobacter oryzae RF3
74%
Delftia acidovorans I1_6
69%
99%
Caenimonas koreensis EMB320
99%
Curvibacter delicatus NBRC 14919
Extensimonas vulgaris S4
72%
Burkholderia cepacia DSM 7288
100%
Oxalobacter formigenes ATCC 35274
99%
Sutterella stercoricanis CCUG 47620
100%
Sutterella wadsworthensis WAL 9799
Parasutterella secunda YIT
Alcaligenes faecalis ATCC 8750
100%
Bacillus licheniformis DSM 13
Escherichia coli K-12 substr. MG1655 strain K-12
Comamonadaceae
Burkholderiales

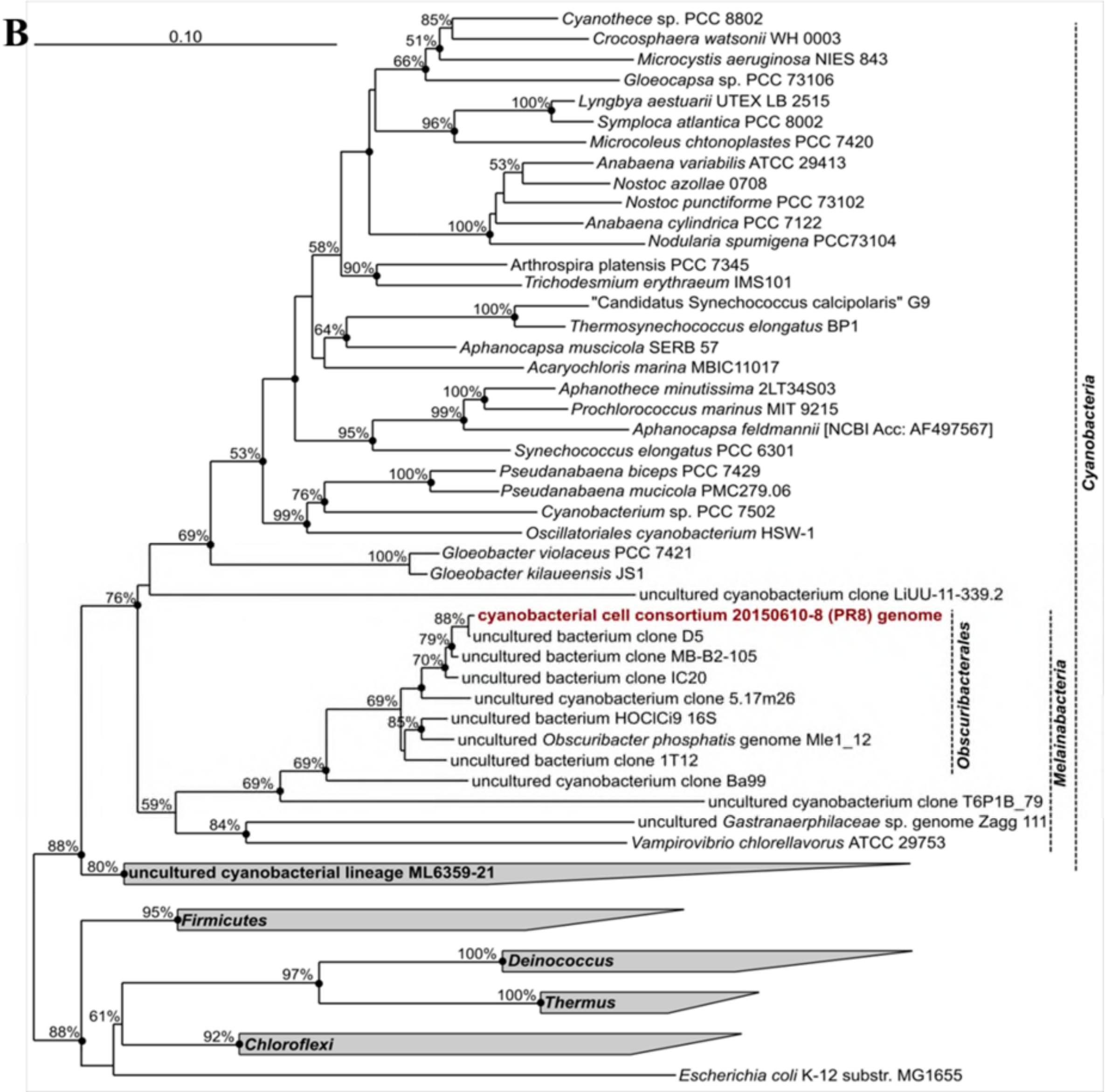

Figure S6. (A) Phylogenetic tree of three *Pelomonas* spp. P728-5, P709-11 and P610-5 isolated in this study. **(B)** Phylogenetic tree of a novel *Cyanobacteria* spp. isolated in this study, which is close to an uncultured *Melainiabacteria* spp..

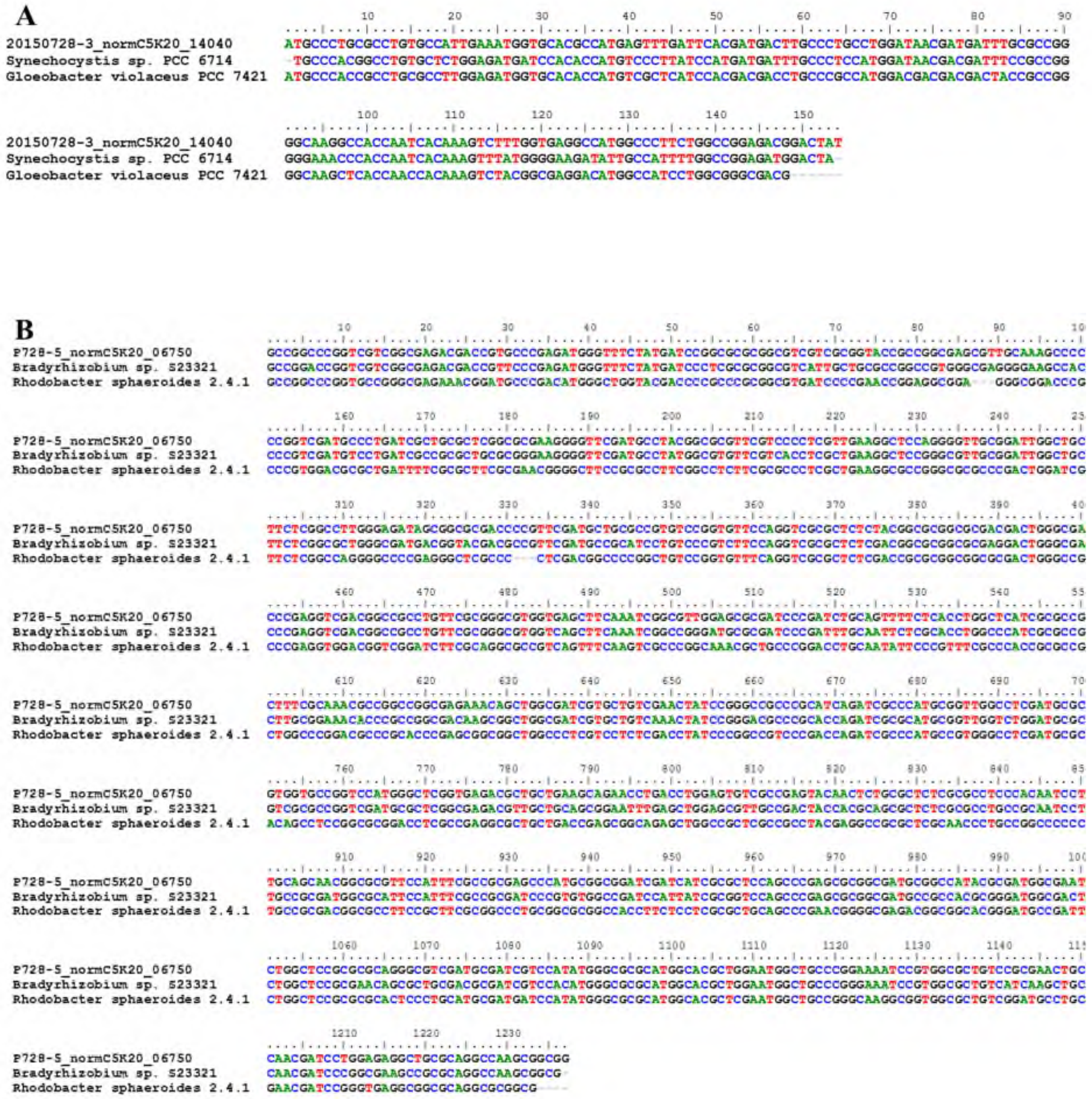
A
20150728-3_normC5K20_14040
Synechocystis sp. PCC 6714
Gloeobacter violaceus PCC 7421
B
P728-5_normC5K20_06750
Bradyrhizobium sp. S23321
Rhodobacter sphaeroides 2.4.1

C

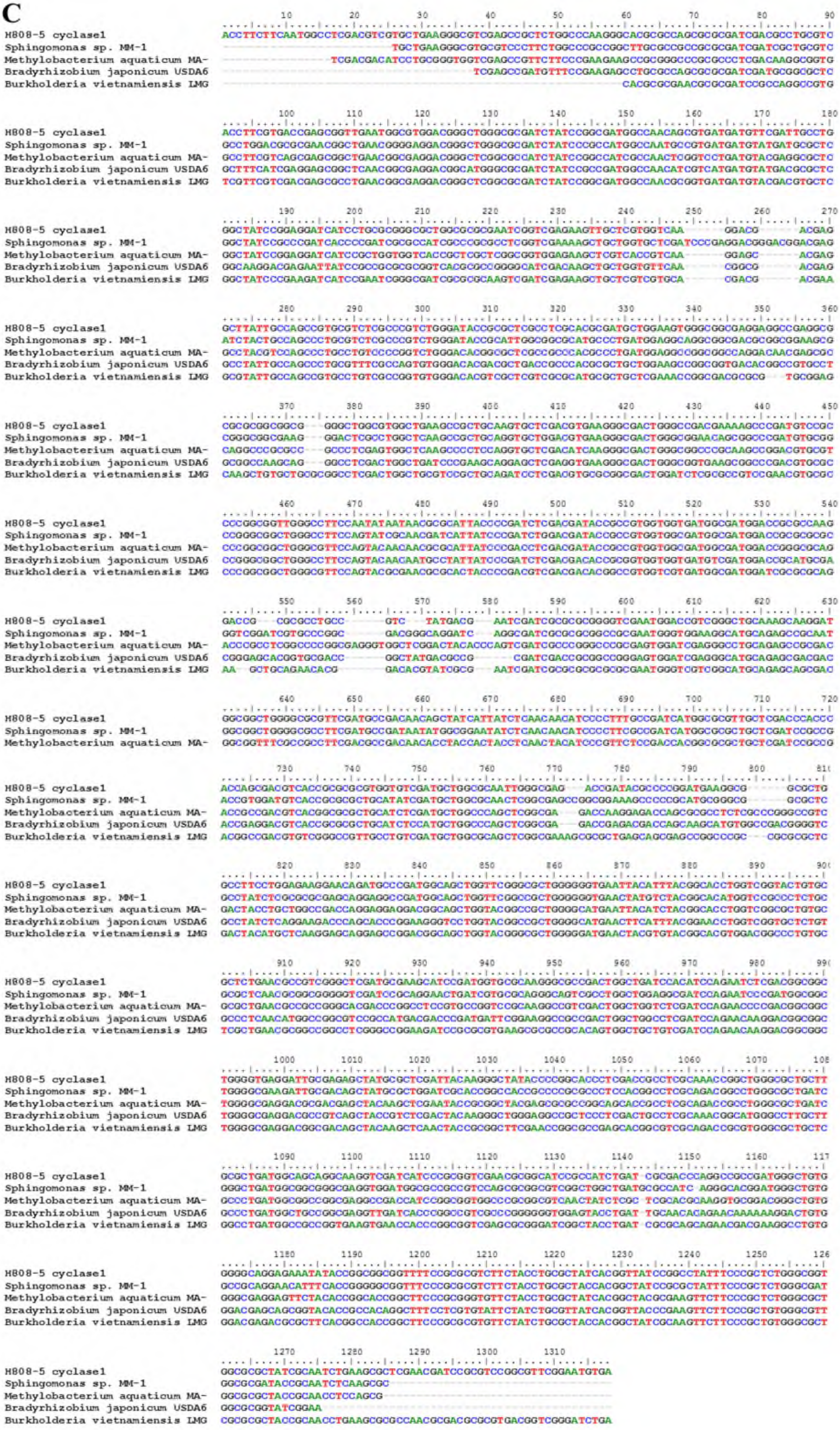
H808-5 cyclase1
Sphingomonas sp. MM-1
Methylobacterium aquaticum MA-
Bradyrhizobium japonicum USDA6
Burkholderia vietnamiensis LMG

D

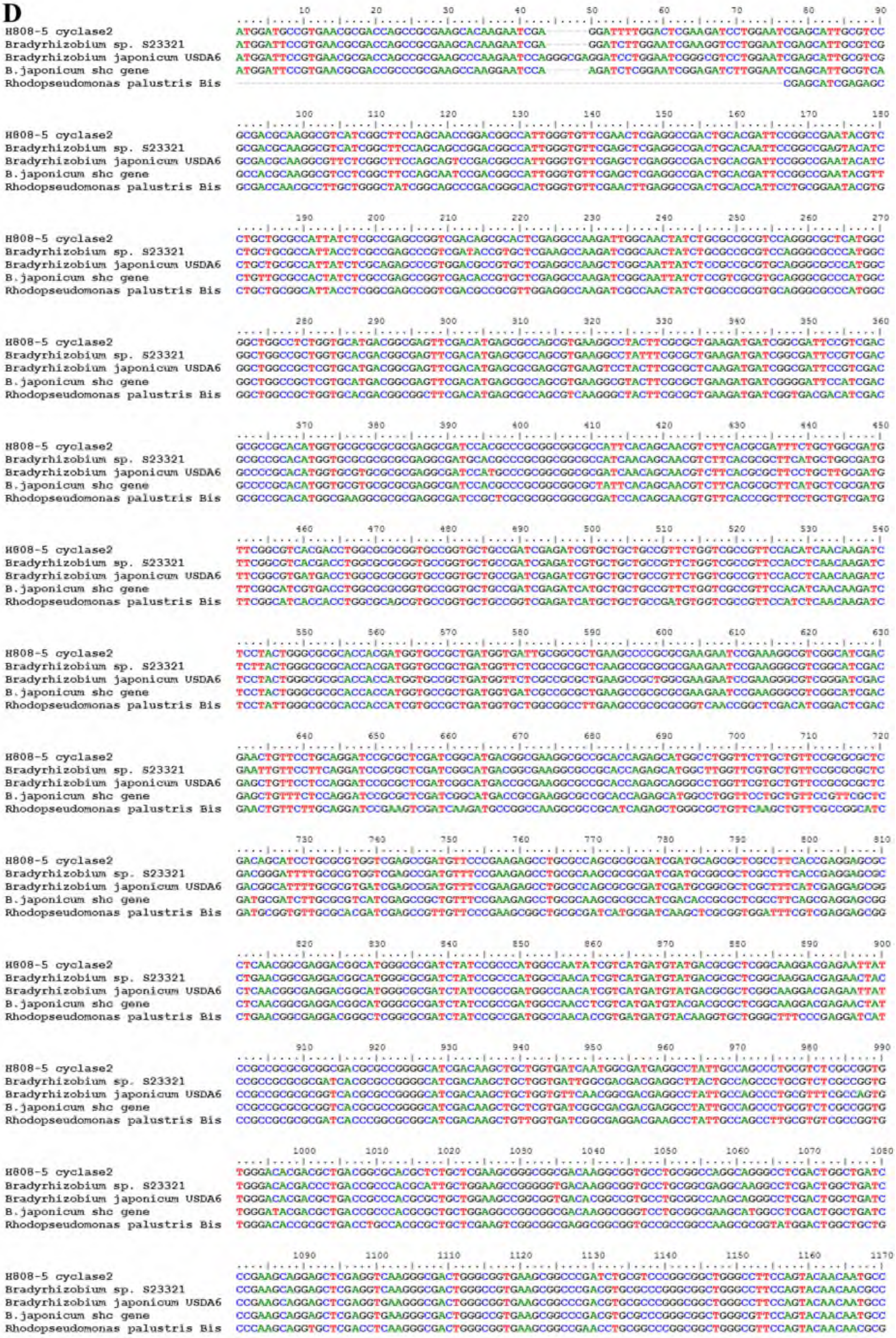
H808-5 cyclase2
Bradyrhizobium sp. S23321
Bradyrhizobium japonicum USDA6
B.japonicum shc gene
Rhodopseudomonas palustris Bis

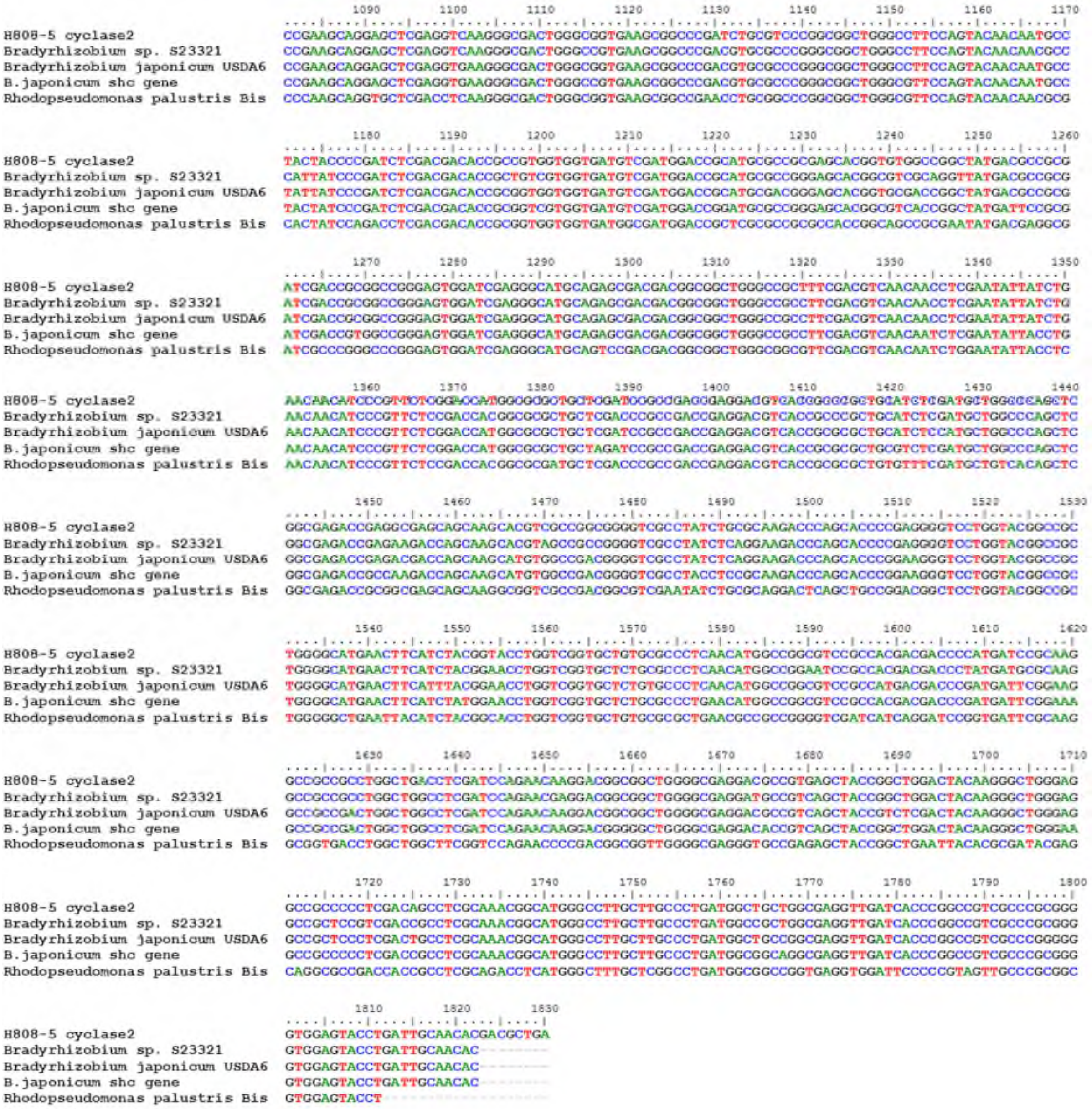

Figure S7. (A) The partial gene encoding CrtE from *Bradyrhizobium* spp. B728-3 (1 cell) is novel and related to *crtE* genes from Cyanobacteria *Synechocystis* spp. and *Gloeobacter* spp. according to BLAST analysis. **(B)** The gene encoding CobN/magnesium chelatase from *Pelomonas* spp. P728-5 (1 cell) and its alignment with homologous genes from *Bradyrhizobium* spp. and *Rhodobacter sphaeroides*. **(C)** *shc* gene1 encoding squalene-hopene cyclase1 isolated from *Halomonas* spp. *H808-5* (1 cell) and its alignment with other *shc* genes. **(D)** *shc* gene2 encoding squalene-hopene cyclase2 isolated from *Halomonas* spp. *H808-5* (1 cell) and its alignment with other *shc* genes.

Tables

Table S1: Pulsed laser power comparison for cell isolation

	Raman activated cell ejection	Zeiss PALM laser microdissection	Leica laser microdissection
**Laser**	532 nm	337/355 nm	349/355 nm
**Pulsed energy (μJ)**	4.3	>270/100	70, 120
**Pulse duration**	60 μs	1-3 ns	1 ns
**Pulse frequency**	50-10,000 kHz	1-100 Hz	10-5000 Hz
**Pulse width**	0.95 ns	0.1 ms	<4 ns
**Peak power (kW)**	1.1-4.2	83.5-93.9	112.7
**Maximal laser on mirrors***	0.89-3.4 mJ/cm^2		

*power on the mirrors in the Raman instrument should be less than 400 mJ/cm^2 according to the manufacturer's instructions.

Table S2: Primers used in this study

Description	Name	Sequence (5'-3')	Refs.
**16S rRNA long**	63F	CAGGCCTAACACATGCAAGTC	(1)
	1387R	GGGCGGWGTGTACAAGGC	(1)
**16S rRNA short**	338F	ACTCCTACGGGAGGCAGC	(2)
	530R	GTATTACCGCGGCTGCTG	(2)
***gfp* gene**	GFP_F	CGATTTAACGCCTAGAATTCGGATCCTAAT	(3)
	GFP_R	ACTCTAGATCTTTAGTATAGTTCATCCATG	(3)
***usp*A gene**	uspA_f	CCGATACGCTGCCAATCAGT	(4)
	uspA_r	ACGCAGACCGTAGGCCAGAT	(4)
